# Supplementary material for: Conversion and Obsessive–Phobic Symptoms Predict IL-33 and IL-28A Levels in Individuals Diagnosed with COVID-19
Source: Brain Sci. 2023 Aug 31;13(9):1271. doi: 10.3390/brainsci13091271 (PMC10526257; doi:10.3390/brainsci13091271)
Supplement: Supplementary file 1 [file brainsci-13-01271-s001.zip › Table S1.pdf]

Table S1. Distribution of raw data by ADS.

	BS	VS	CS	OPS	DS
Valid	50	51	51	48	50
Missing	2	1	1	4	2
Mean	3.08	5.27	1.02	3.42	1.68
Median	2.00	4.00	.00	2.00	1.00
Mode	1	3	0	1	0
S.D.	2.54	3.39	1.35	3.13	2.17
Skewness	.663	.704	1.541	.897	1.628
Kurtosis	-.547	-.529	2.645	-.246	2.011
Minimum	0	1	0	0	0
Maximum	9	14	6	11	8

* Multiple modes exist. The smallest value is shown.

Legend:

Standart Deviation-S.D., Basic syndrome-BS, Vegetative syndrome-VS, Conversion syndrome-CS, Obsessive-phobic syndrome-OPS, Depressive syndrome-DS
