# Supplementary material for: Conversion and Obsessive–Phobic Symptoms Predict IL-33 and IL-28A Levels in Individuals Diagnosed with COVID-19
Source: Brain Sci. 2023 Aug 31;13(9):1271. doi: 10.3390/brainsci13091271 (PMC10526257; doi:10.3390/brainsci13091271)
Supplement: Supplementary file 1 [file brainsci-13-01271-s001.zip › Table S2.pdf]

Table S2. Internal consistency of ADS.

Subscales	Cronbach's Alpha	No. of items
Basic syndrome	0.770	9
Vegetative syndrome	0.781	16
Conversion syndrome	0.636	7
Obsessive-phobic syndrome	0.830	13
Depressive syndrome	0.825	8
Anxiety-Depressive Scale	0.934	65
