# Supplementary material for: Conversion and Obsessive–Phobic Symptoms Predict IL-33 and IL-28A Levels in Individuals Diagnosed with COVID-19
Source: Brain Sci. 2023 Aug 31;13(9):1271. doi: 10.3390/brainsci13091271 (PMC10526257; doi:10.3390/brainsci13091271)
Supplement: Supplementary file 1 [file brainsci-13-01271-s001.zip › Table S3.pdf]

Table S3. Differences by gender in ADS between persons diagnosed with COVID-19 (Independent-Samples T-Test) (N=52).

Scales	Groups	N	Mean	SD	t	df	p
BS	Women	25	3.28	2.46	.553	48	.291
	Men	25	2.88	2.65			
VS	Women	26	6.31	3.51	2.315	49	.012
	Men	25	4.20	2.96			
CS	Women	26	1.15	1.49	.721	49	.237
	Men	25	.88	1.20			
OPS	Women	24	3.54	3.02	.274	46	.393
	Men	24	3.29	3.29			
DS	Women	25	1.68	1.97	.000	48	.500
	Men	25	1.68	2.39			
ADS	Women	24	15.79	10.96	1.117	46	.135
	Men	24	12.33	10.49			
