# Supplementary material for: Conversion and Obsessive–Phobic Symptoms Predict IL-33 and IL-28A Levels in Individuals Diagnosed with COVID-19
Source: Brain Sci. 2023 Aug 31;13(9):1271. doi: 10.3390/brainsci13091271 (PMC10526257; doi:10.3390/brainsci13091271)
Supplement: Supplementary file 1 [file brainsci-13-01271-s001.zip › Table S4.pdf]

Table S4. Measures of central tendency and dispersion of interleukins values.

	IL-10/ pg/ml	IL-28A/ ng/l	IL-33/ ng/l
Mean	339.687	61.40852	165.05279
Std. Error of Mean	22.3888	13.043588	35.024510
Std. Deviation	159.8878	94.058650	252.565337
