# Supplementary material for: Conversion and Obsessive–Phobic Symptoms Predict IL-33 and IL-28A Levels in Individuals Diagnosed with COVID-19
Source: Brain Sci. 2023 Aug 31;13(9):1271. doi: 10.3390/brainsci13091271 (PMC10526257; doi:10.3390/brainsci13091271)
Supplement: Supplementary file 1 [file brainsci-13-01271-s001.zip › Table S5.pdf]

Table S5. Correlations between ADS and interleukins values in persons diagnosed with COVID-19 (N=52).

ADS		IL-10	IL-28A	IL-33
BS	Pearson Correlation	.001	-.271	.274
	Sig. (2-tailed)	.993	.057	.054
VS	Pearson Correlation	.101	-.100	.061
	Sig. (2-tailed)	.487	.486	.673
CS	Pearson Correlation	.057	-.032	.006
	Sig. (2-tailed)	.692	.824	.966
OPS	Pearson Correlation	-.164	-.239	.289*
	Sig. (2-tailed)	.270	.102	.046
DS	Pearson Correlation	-.067	-.218	.120
	Sig. (2-tailed)	.648	.128	.408

* Correlation is significant at the 0.05 level (2-tailed).
