# Supplementary figures and images for: Conversion and Obsessive–Phobic Symptoms Predict IL-33 and IL-28A Levels in Individuals Diagnosed with COVID-19
Source: Brain Sci. 2023 Aug 31;13(9):1271. doi: 10.3390/brainsci13091271 (PMC10526257; doi:10.3390/brainsci13091271)

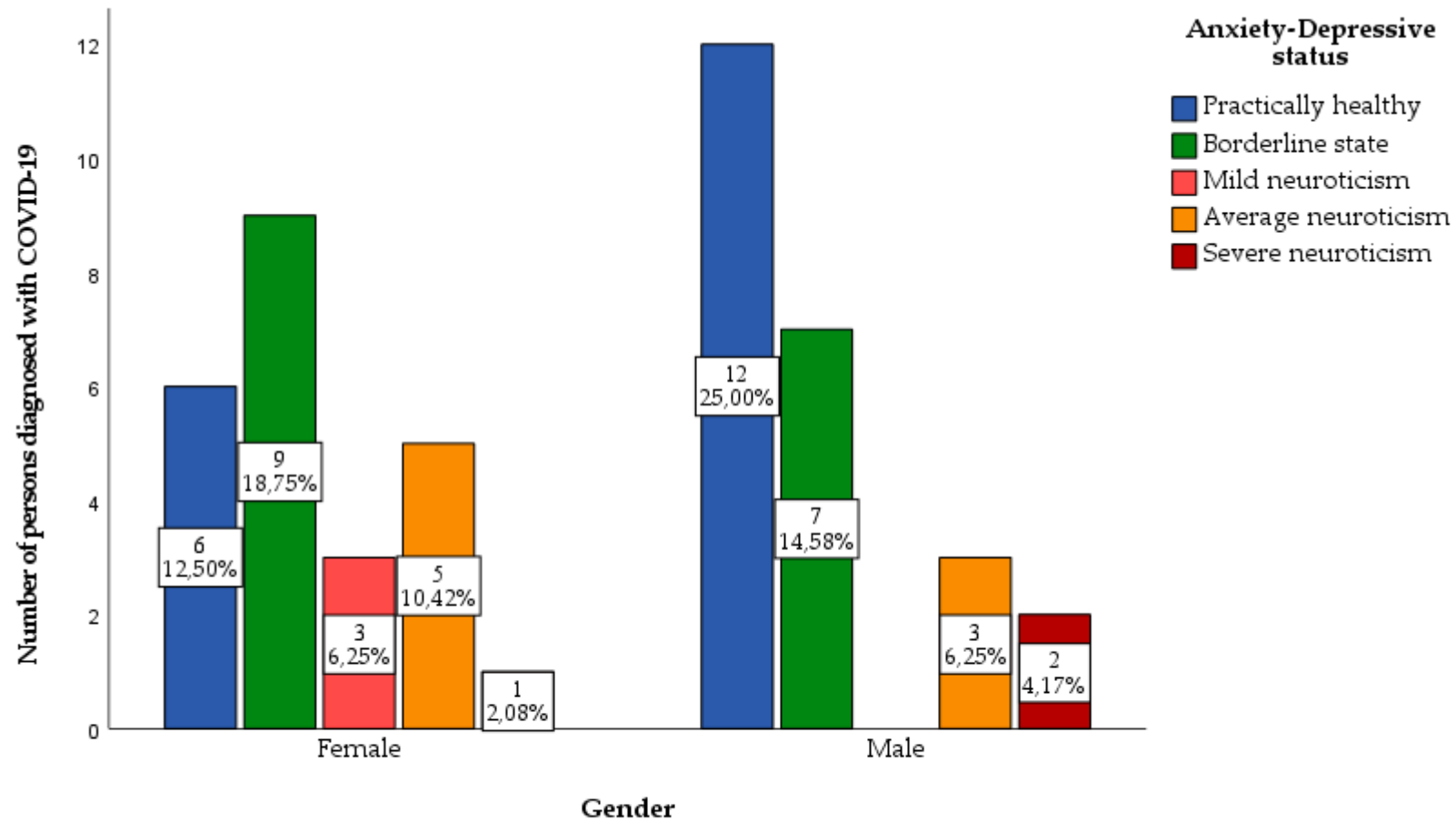

**Figure S1.** Anxiety-Depressive status of persons diagnosed with COVID-19.

Supplement: Supplementary file 1 [file brainsci-13-01271-s001.zip › Figure S1.pdf]

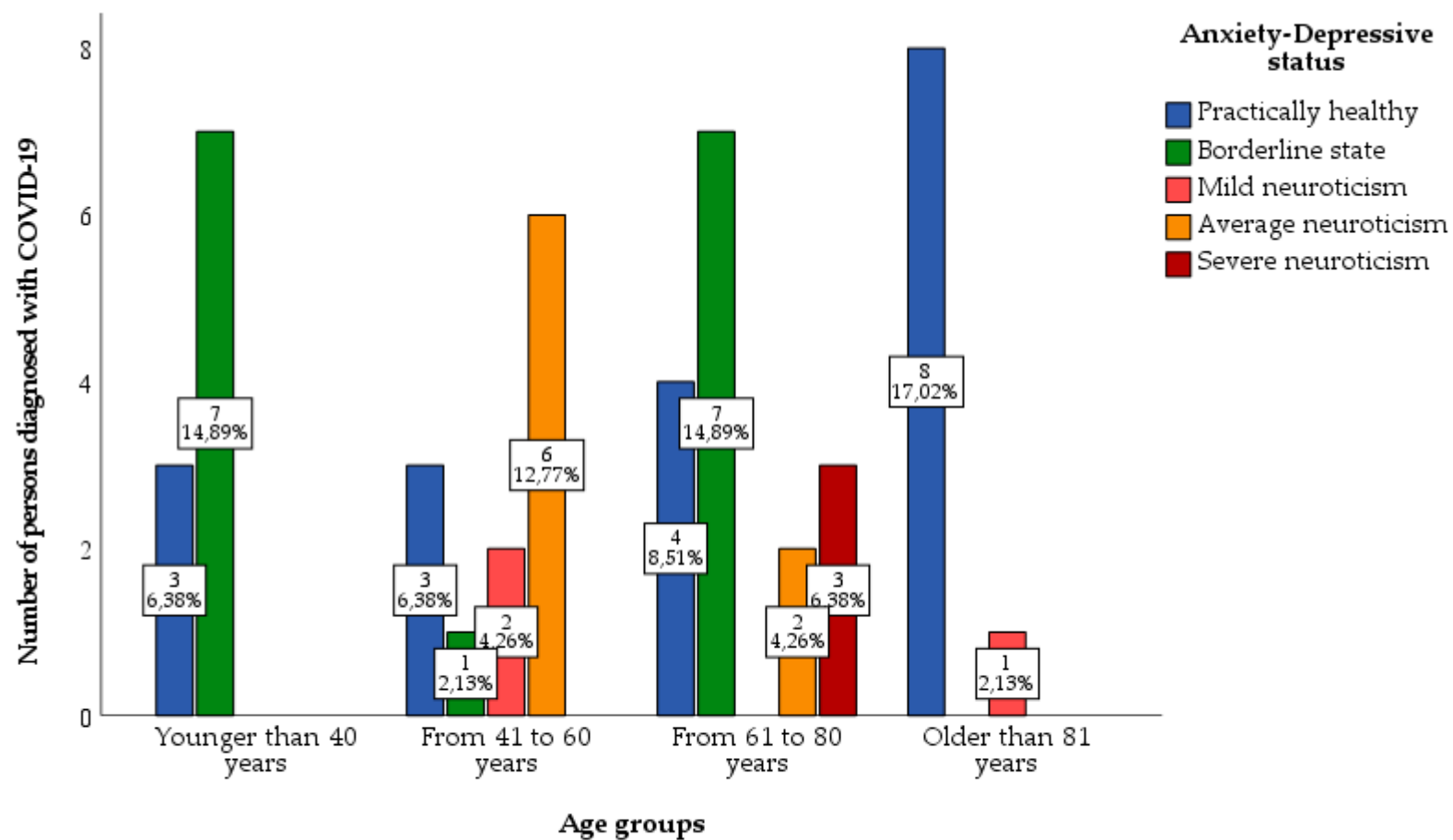

**Figure S2.** Anxiety-Depressive status in persons diagnosed with COVID-19 in different age groups.

Supplement: Supplementary file 1 [file brainsci-13-01271-s001.zip › Figure S2.pdf]

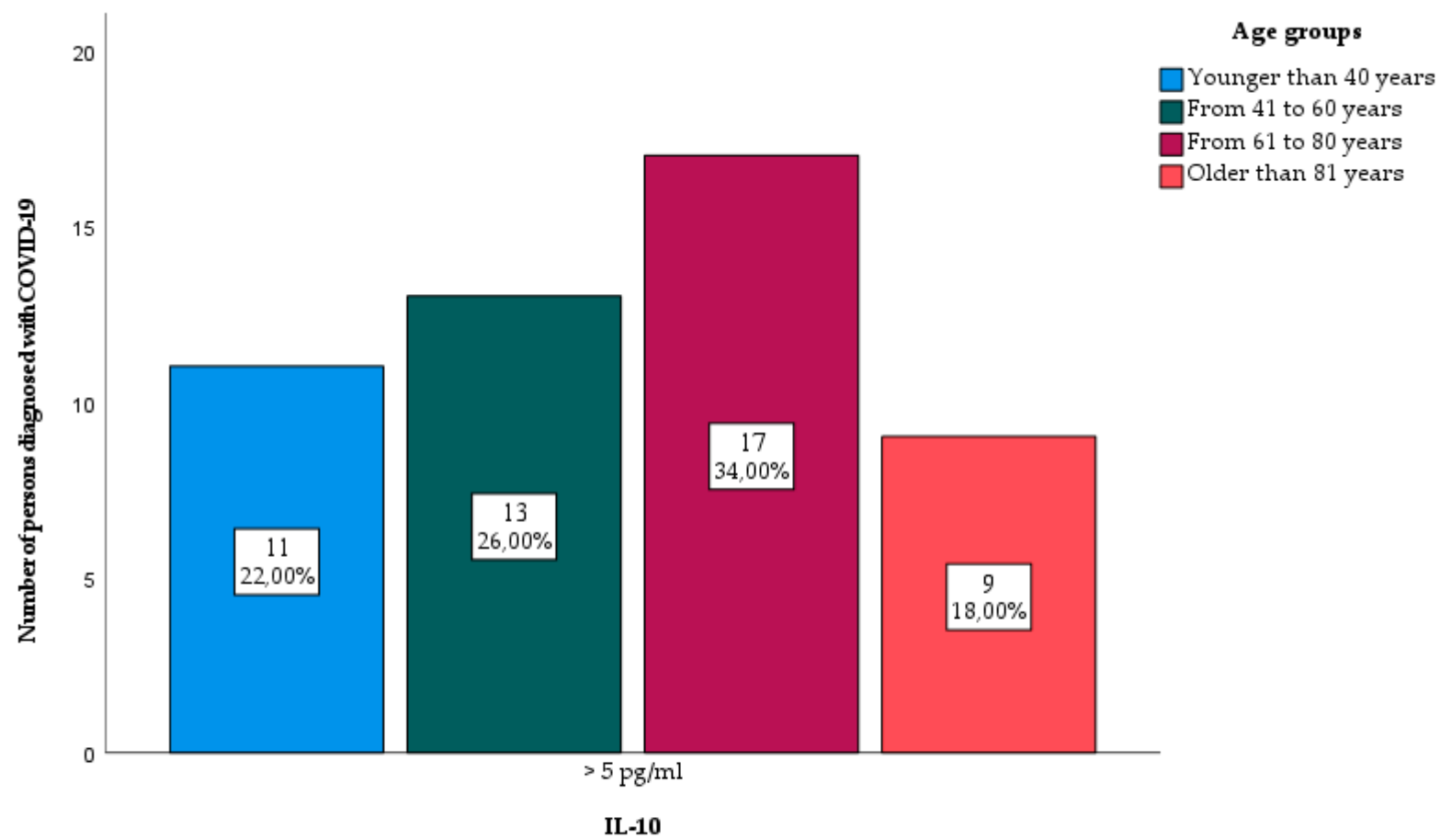

**Figure S3.** Distribution of IL-10 values in different age groups

Supplement: Supplementary file 1 [file brainsci-13-01271-s001.zip › Figure S3.pdf]

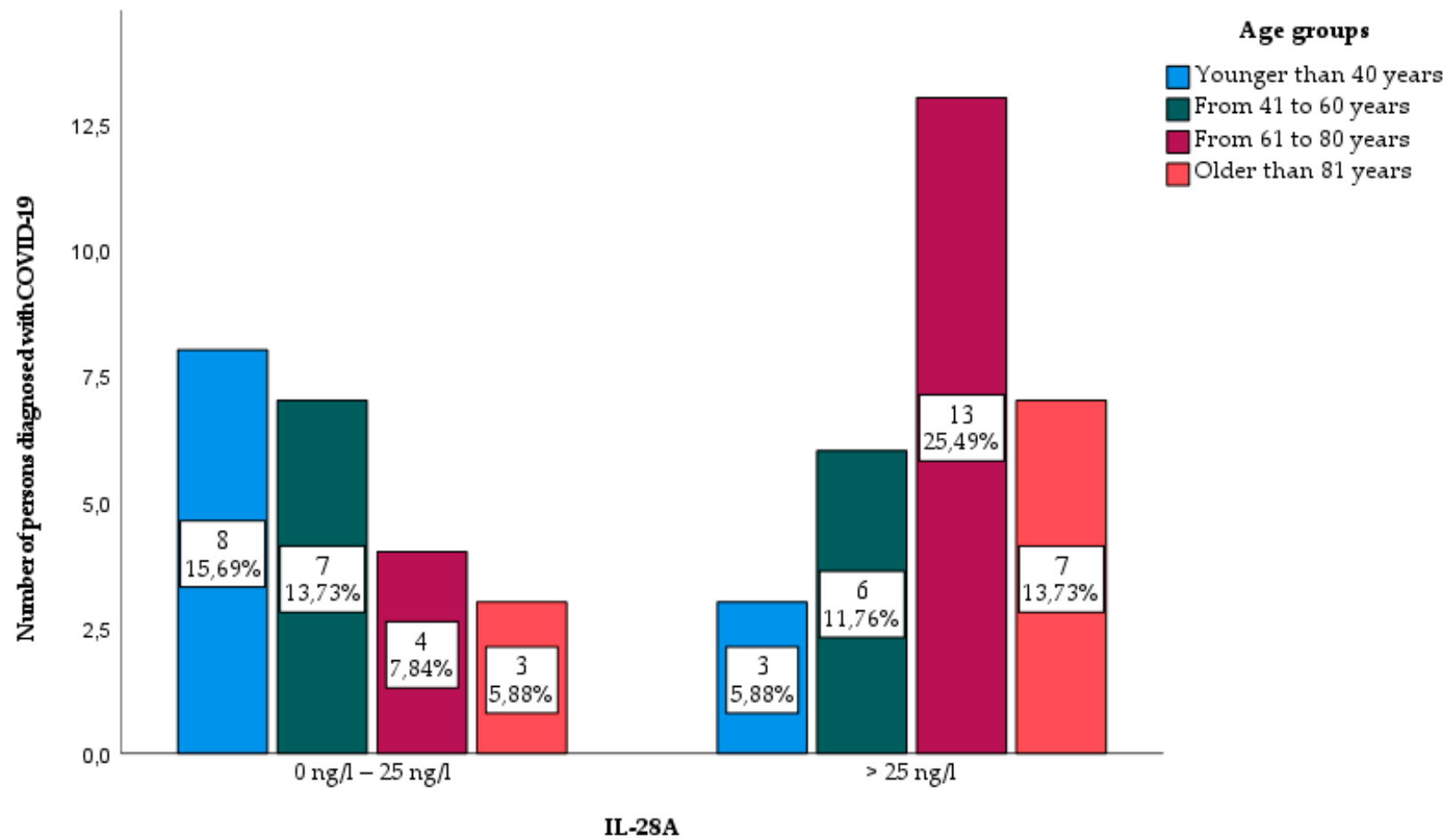

**Figure S4.** Distribution of IL-28A values in different age groups

Supplement: Supplementary file 1 [file brainsci-13-01271-s001.zip › Figure S4.pdf]

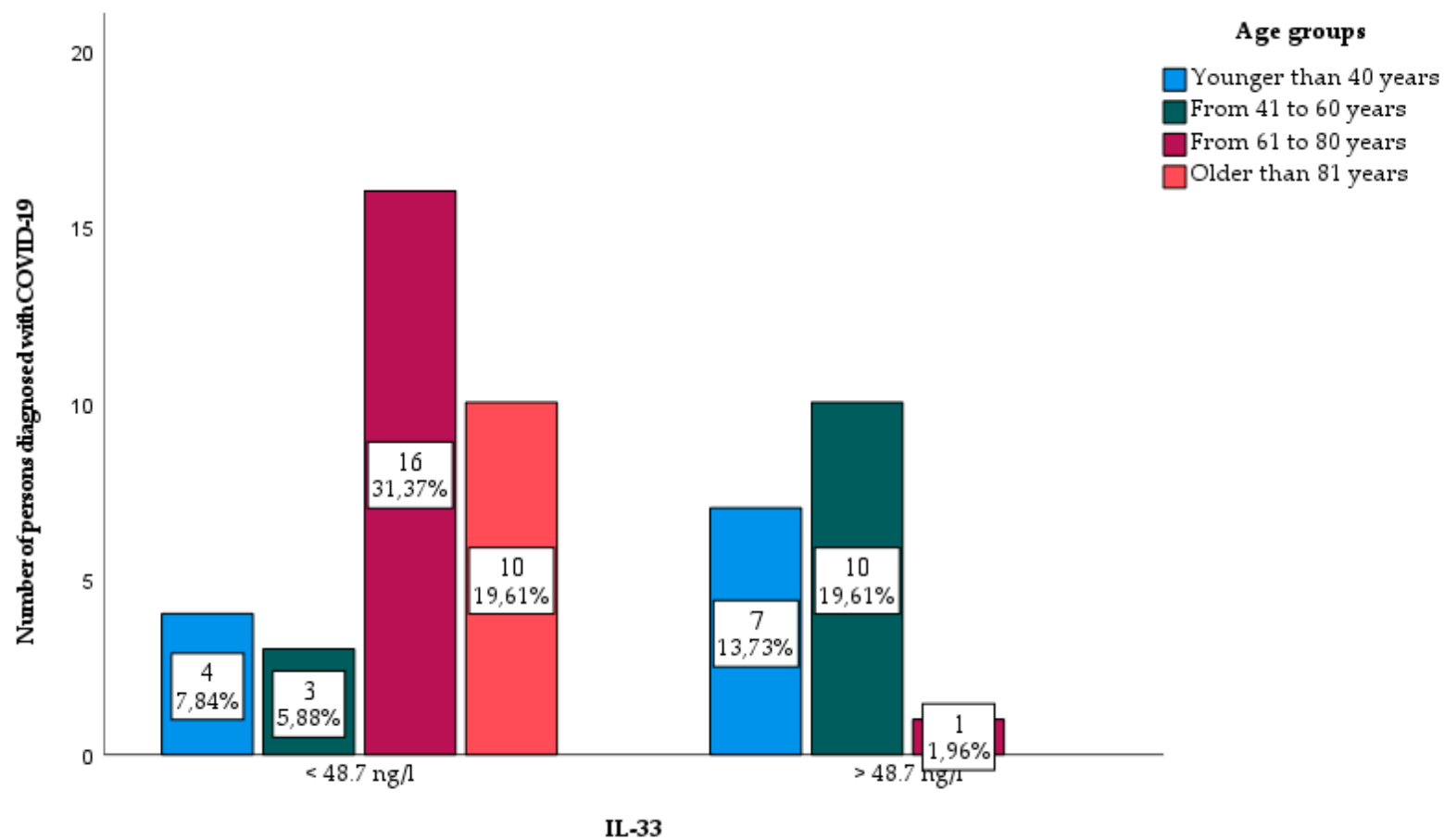

**Figure S5.** Distribution of IL-33 values in different age groups

Supplement: Supplementary file 1 [file brainsci-13-01271-s001.zip › Figure S5.pdf]
